# *Mycobacterium tuberculosis* AtsG (Rv0296c), GlmU (Rv1018c) and SahH (Rv3248c) Proteins Function as the Human IL-8-Binding Effectors and Contribute to Pathogen Entry into Human Neutrophils

**DOI:** 10.1371/journal.pone.0148030

**Published:** 2016-02-01

**Authors:** Bozena Dziadek, Anna Brzostek, Marcin Grzybowski, Marek Fol, Agnieszka Krupa, Jakub Kryczka, Przemyslaw Plocinski, Anna Kurdowska, Jaroslaw Dziadek

**Affiliations:** 1 Department of Immunoparasitology, Faculty of Biology and Environmental Protection, University of Lodz, Lodz, Poland; 2 Institute of Medical Biology, Polish Academy of Sciences, Lodz, Poland; 3 Department of Immunology and Infectious Biology, Faculty of Biology and Environmental Protection, University of Lodz, Lodz, Poland; 4 Department of Cellular and Molecular Biology, The University of Texas Health Science Center at Tyler, Tyler, Texas, United States of America; 5 Laboratory of RNA Biology and Functional Genomics, Institute of Biochemistry and Biophysics, Polish Academy of Sciences, Warsaw, Poland; Public Health Research Institute at RBHS, UNITED STATES

## Abstract

*Mycobacterium tuberculosis* is an extremely successful intracellular pathogen that has evolved a broad spectrum of pathogenic mechanisms that enable its manipulation of host defense elements and its survival in the hostile environment inside phagocytes. Cellular influx into the site of mycobacterial entry is mediated by a variety of chemokines, including interleukin-8 (IL-8), and the innate cytokine network is critical for the development of an adaptive immune response and infection control. Using affinity chromatography, liquid chromatography electrospray ionization tandem mass spectrometry and surface plasmon resonance techniques, we identified *M*. *tuberculosis* AtsG arylsulphatase, bifunctional glucosamine-1-phosphate acetyltransferase and N-acetylglucosamine-1-phosphate uridyl transferase (GlmU) and S-adenosyl-L-homocysteine hydrolase (SahH) as the pathogen proteins that bind to human IL-8. The interactions of all of the identified proteins (AtsG, GlmU and SahH) with IL-8 were characterized by high binding affinity with K_D_ values of 6.83x10^-6^ M, 5.24x10^-6^ M and 7.14x10^-10^ M, respectively. Furthermore, the construction of *Mtb* mutant strains overproducing AtsG, GlmU or SahH allowed determination of the contribution of these proteins to mycobacterial entry into human neutrophils. The significantly increased number of intracellularly located bacilli of the overproducing *M*. *tuberculosis* mutant strains compared with those of “wild-type” *M*. *tuberculosis* and the binding interaction of AtsG, GlmU and SahH proteins with human IL-8 may indicate that these proteins participate in the modulation of the early events of infection with tubercle bacilli and could affect pathogen attachment to target cells.

## Introduction

Tuberculosis, an aerosol-transmitted infectious disease caused by the bacterial intracellular pathogen *Mycobacterium tuberculosis* (*Mtb*), is second only to HIV/AIDS in infectious disease mortality worldwide. According to the World Health Organization (WHO), in 2013, there were an estimated 9 million new tuberculosis cases, and 1.5 million people died from the disease [1]. The effective treatment and control of tuberculosis is currently a serious challenge to global health efforts. The situation is really alarming due to the emergence and rapid spread of multi-drug resistant (MDR) [2–4], extensively-drug resistant (XDR) [3–5] and totally drug-resistant (TDR) [6–8] strains of *Mtb* that cannot be cured with standard anti-tuberculosis drugs. This unexpected increase in the drug resistance of mycobacteria has become a major incentive for undertaking extensive research to develop new anti-tuberculosis therapeutic weapons. It is obvious that such studies cannot be conducted without fully understanding the metabolic pathways of tubercle bacilli and the broad spectrum of interactions between the pathogen and components of the host immune response. Advances in these fields are a prerequisite for the search and precise selection of new molecular targets for modern drugs that could contribute to the effective control of tuberculosis.

As a very successful pathogen *Mtb* triggers a number of strategies to circumvent the hostile host defense and to ensure the intracellular survival and replication of the pathogen within macrophages, the main effector cells of innate immunity. These strategies rely mainly on molecular interactions of mycobacterial cell components and secretory products with specific targets engaged in the host immune response. Interestingly, after inhalation into the respiratory tract, tubercle bacilli gain entry into macrophages by exploiting a wide range of the host’s soluble and cell surface pattern recognition molecules (PRMs). Mycobacterial mannosylated lipoarabinomannan (ManLAM) has been proven to be a key pathogen-associated molecular pattern (PAMP) involved in the binding of different C-type lectins (collectins), such as mannose-binding lectin (MBL), pulmonary surfactant proteins A (SP-A) and D (SP-D), and collectin-11 (CL-K1), which serve as PRMs [9–12]. However, other *Mtb* ligands for SP-A, including lipomannan [13], a 60-kDa glycoprotein [14], and the surface alanine- and proline-rich antigenic glycoprotein Apa [15], have also been identified. In addition to collectins, other host ManLAM target molecules, ficolin-3 and fibronectin, have been described [9,10,16]. Both ficolin-3 and fibronectin function as opsonins and support the mycobacterial invasion of macrophages and airway epithelial cells, respectively. The successful infection of macrophages by *Mtb* is also enhanced by bacterial interaction with complement component C3. The mycobacterial heparin-binding hemagglutinin (Hbha) protein has been identified as a complement fragment C3-binding surface molecule of tubercle bacilli [17]. The C3-opsonized mycobacteria are selectively recognized by the macrophage “Trojan horse” complement receptors CR1, CR3 and CR4, providing a C3-dependent entry pathway into alveolar mononuclear phagocytes [17,18].

Modulation of the internalization process is not the only strategy that contributes to the virulence of *Mtb*. An arsenal of tubercle bacilli cell wall and secretory effectors, e.g., ManLAM, lipoprotein LpqH and acid phosphatase SapM, protein tyrosine phosphatase A (PtpA), eukaryotic-like serine/threonine protein kinase (PknG), nucleotide diphosphate kinase (NadK), lipoamide dehydrogenase (LpdC), MTSA-10 antigen, secretory proteins of the ESX-1 system, respectively, interact with specific target substrates or pathways in the host [19,20]. All of these complex virulence strategies are utilized to achieve impairment of the host antimicrobial mechanisms and to establish an ideal niche within macrophages. Inhibition of the major immune functions of macrophages in turn contributes to the disruption of the development of the appropriate powerful innate and adaptive cellular immune responses.

In addition to the above-mentioned *Mtb* virulence mechanisms, another intriguing ability that influences the fate of pathogen infection is the interaction of the mycobacteria with the cytokine network. Among the many cytokines synthesized during the course of *Mtb* infection, interleukin-8 (IL-8) is one of the most important for the establishment of the proper immune response. Interleukin-8 belongs to the family of CXC chemokines and functions as a chemoattractant and activator of different subsets of leukocytes. The main producers of this chemokine during tuberculosis are infected monocytes and alveolar macrophages as well as neutrophils and nonimmune pulmonary epithelial cells [21–26]. In addition to being responsible for neutrophil sequestration in *Mtb*-infected lungs, IL-8 enhances the mycobactericidal properties of these cells [27]. Additionally, IL-8 contributes to the recruitment of monocytes and T lymphocytes to the pulmonary compartment and has been found to be absolutely required for granuloma formation [24,28,29]. Nevertheless, the exact role of IL-8 in the pathogenesis of tuberculosis is not fully understood because the accumulation of neutrophils in the airway can, on the one hand, contribute to eradication of tubercle bacilli and development of dendritic and T cell responses and, on the other hand, lead to pathogen dissemination and pulmonary tissue damage through the release of cytotoxic molecules [30]. Moreover, some conflicting data suggest that a high plasma concentration of IL-8 is correlated with either mortality from tuberculosis or positive clinical prognosis in patients infected with *Mtb* [31].

In a previous study [28], we found that tubercle bacilli are capable of binding to human IL-8. Additionally, we demonstrated that the noted host IL-8-pathogen interaction contributes to the increased mycobactericidal properties of macrophages and neutrophils. In the present paper, we identify mycobacterial AtsG (Rv0296c; arylsulfatase), GlmU (Rv1018c; bifunctional glucosamine-1-phosphate acetyltransferase and N-acetylglucosamine-1-phosphate uridyl transferase) and SahH (Rv3248c; S-adenosyl-L-homocysteine hydrolase) proteins as candidate ligands targeting human IL-8. Furthermore, we also demonstrate the involvement of the identified mycobacterial IL-8-binding proteins in pathogen entry into human neutrophils and show the participation of IL-8 in the phenomenon of *Mtb* opsonization.

## Material and methods

### Mycobacterial strains and growth conditions

Mycobacterial “wild-type” and mutant strains were grown in roller bottles in Middlebrook 7H9 broth supplemented with BBL^TM^ Middlebrook 10% Oleic Albumin Dextrose Catalase (OADC) Enrichment (Becton Dickinson), 0.05% Tween80 (Sigma) and 50 ng/ml tetracycline (mutant strains only) (Sigma) until reaching an optical density of OD_600_ = 1.0 (4–6 days). The bacterial suspensions were then aliquoted and stored at -85°C. After one week, randomly selected samples of frozen mycobacteria were thawed, and the number of live bacterial cells was evaluated by determination of the colony forming units (CFUs).

### Preparation of *Mtb* whole-cell extract

The *Mycobacterium tuberculosis* H37Rv strain was cultured as described above, and once reaching an optical density of 1.0 at λ = 600 nm, the bacterial cells were collected by centrifugation and resuspended in cold 1X PEN lysis buffer at pH 6.5 (3X PEN: 300 mM Na_2_HPO_4_, 200 mM NaH_2_PO_4_, 450 mM NaCl, and 0.3 mM EDTA) according to the protocol described by Heinz et al. [32]. After the addition of BigB Lysing Matrix (MP Biomedicals) the mycobacterial cells were disrupted using an ultrasound disintegrator (FastPrep-24, MP Biomedicals) by the application of three 20-s cycles (6 m/s) performed at 3-min intervals on ice followed by a 30-min incubation in POP05 buffer (1X PEN buffer containing 0.5% (m/v) n-octylpolyoxyethylene, OPOE; Sigma) at room temperature with gentle rocking. Finally, the prepared mycobacterial cell lysates were centrifuged at 16,000 x g and 4°C for 30 min, aliquoted and stored at 4°C for further applications. The protein concentration in the whole-cell extracts was determined using the Bradford Reagent (Sigma) following the manufacturer’s instructions.

### Purification of *Mtb* human IL-8-binding proteins

Human IL-8-binding proteins of *Mtb* were purified by affinity chromatography using a MicroLink Protein Coupling Kit (Thermo Scientific Pierce) according to the protocol recommended by the manufacturer. In brief, the AminoLink Plus Coupling Resin was coupled with 100 μg of recombinant human IL-8 (Gibco) and, after blocking of the remaining active binding sites, incubated overnight with 500 μg of mycobacterial whole-cell proteins at 4°C with gentle end-over-end rotation. To eliminate unspecific interactions of the coupled ligand with the mycobacterial proteins, the resin was washed with Washing Buffer (0.5 M NaCl; Sigma) containing 0.05% Tween20 (Sigma) and, the bound *Mtb* proteins were then eluted by three incubations of the chromatography resin with 100 μl of Elution Buffer at pH 2.8. The acidic eluates were then immediately neutralized by the addition of 5 μl of 1 M Tris (pH 9.0), and the eluted proteins were precipitated by overnight incubation at -20°C with cold acetone at a final concentration of 25% (v/v). The precipitates were centrifuged at 16,000 x g and 4°C, dried at room temperature to remove any trace of acetone and finally resolved in 40 μl of cold PBS.

### Analysis of the affinity chromatography-purified mycobacterial proteins

The affinity chromatography-purified proteins of *Mtb* that presumably bind to human IL-8 were analyzed with a standard SDS-PAGE electrophoresis technique performed under reducing conditions on 4–20%-gradient Precise Tris-Glycine Gels (Thermo Scientific Pierce) in the Mini-Protean System (Bio-Rad). The reduced protein samples were prepared by heating at 37°C for 20 min in 5x sample buffer containing SDS and 2-mercaptoethanol at final concentrations of 4% (w/v) and 5% (v/v), respectively. Before each analysis, the separated proteins were stained using a commercial Zinc Reversible Stain Kit (Thermo Scientific Pierce), which provides highly sensitive detection of even 0.25 ng of protein per band.

### Identification of *Mtb* proteins binding human IL-8

Affinity chromatography-purified human IL-8-binding mycobacterial proteins were identified using the liquid chromatography electrospray ionization tandem mass spectrometry (LC/MS/MS) technique. Prior to sequencing, the proteins were separated by SDS-PAGE electrophoresis, cut from the gel and then sent to the Institute of Biochemistry and Biophysics Polish Academy of Sciences (Warsaw, Poland) for analysis.

The gel bands were reduced with DTT, alkylated with iodoacetamide and digested with trypsin according to standard procedures [33]. The resulting peptide mixtures were recovered from the gel pieces and applied to a nano-HPLC RP-18 column (Waters) using an acetonitryle gradient in the presence of trifluoroacetic acid. The column was directly linked to the ion source by running blank runs in between each sample. The Orbitrap was operated in a data-dependent mode to automatically switch between MS and LTQ-MS/MS acquisition.

The MaxQuant (v1.3.0.5) computational proteomics platform was used to process raw MC files [34]. Integrated Andromeda search engine and *M*. *tuberculosis* protein database (http://tuberculist.epfl.ch/, TubercuList_R25, 4022 protein entries) were used to search against the fragmentation spectra. Carbamidomethylation of cysteines was chosen as a fixed modification in MaxQuant search parameters. N-terminal acetylation and oxidation were set as variable modifications. One percent false discovery rate was applied to all protein and peptide identifications. MaxQuant integrated “common contaminants” database allowed identification of human derived contaminants like skin keratins and trypsin. Contaminants and random protein identifications were excluded from the result file. Proteins considered as valid identifications were identified by at least two peptides.

### Cloning of *Mtb atsG*, *serA*, *sahH* and *glmU* genes

The total genomic DNA of *Mtb* H37Rv strain, *Pfx* DNA polymerase (Invitrogen) and appropriate primer pairs (Table 1) were used for amplification of the *atsG*, *serA*, *sahH* and *glmU* genes. To facilitate sub-cloning into an expression vector, restriction enzyme recognition sites were incorporated into the primer sequences (underlined sequences). All of the PCR products were cloned initially into the pJET1.2/blunt vector (Fermentas) and then, after sequence verification and digestion with NcoI and HindIII (*atsG* gene) or BamHI and HindIII (*serA*, *sahH* and *glmU* genes) endonucleases, sub-cloned into the final pHis.Parallel1 expression vector to enable synthesis of recombinant fusion proteins containing an extra 6-His Tag domain at the N-terminus [35]. The resultant constructs marked pHis-atsG, pHis-serA, pHis-sahH or pHis-glmU were introduced into host *E*. *coli* BL21(DE3) cells to express the relevant *Mtb* recombinant forms of the proteins selected by affinity chromatography as potentially interacting with human IL-8.

**Table 1:** Primer sequences used for PCR amplification of gene sequences.

Amplified region	Primer	Sequence (5’→3’)^a^	Product size [bp]
***Proteins expression in E*. *coli***			
*atsG* sense	atsG/NcoI	GCCATGGATGTGACGAGTGAGCGTGCCACAGG	1398
*atsG* reverse	atsG/HindIII	CAAGCTTACTAGCTGCAGTGTTCGTCGATGCCG	
*glmU* sense	glmU/BamHI	CGGATCCGATGACGTTTCCTGGTGACACCGC	1488
*glmU* reverse	glmU/HindIII	CAAGCTTATCACGGTGTCTGATCAGCGTCGG	
*sahH* sense	sahH/BamHI	CGGATCCGATGACCGGAAATTTGGTGACCAAAAATTCG	1488
*sahH* reverse	sahH/HindIII	CAAGCTTATCAGTAGCGGTAGTGGTCCGGCTTG	
*serA* sense	serA/BamHI	CGGATCCGGTGAGCCTGCCTGTTGTGTTGATCG	1587
*serA* reverse	serA/HindIII	CAAGCTTATCACGACAGATCGACAACCTCGAGC	
***Construction of the overproducing mycobacterial strains***			
*atsG* sense	aslA/sBglII KW08Rv	AGATCTGGAGGAAATGTTATGACGAGTGAGCGTGCCACAGG	1398
*atsG* reverse	aslA/rHindIIIKW08Rv	CAAGCTTCTAGCTGCAGTGTTCGTCGATGCC	
*glmU* sense	glmU/sBamHI KW08Rv	AGGATCCGGAGGAAATGTTATGACGTTTCCTGGTGACACCGC	1488
*glmU* reverse	glmU/rHindIII KW08Rv	CAAGCTTTCACGGTGTCTGATCAGCGTCGG	
*sahH* sense	sahH/sBamHI KW08Rv	AGGATCCGGAGGAAAGTTTATGACCGGAAATTTGGTGACCAAAAATTCG	1488
*sahH* reverse	sahH/rHindIII KW08Rv	CAAGCTTCAGTAGCGGTAGTGGTCCGGCTTG	

^a^ The restriction enzyme recognition sites are underlined

### Expression and purification of recombinant rAtsG, rSerA, rSahH and rGlmU proteins in *Escherichia coli*

*Escherichia coli* BL12(DE3) transformed with the pHis-atsG, pHis-serA, pHis-sahH or pHis-glmU recombinant plasmid was grown at 37°C in LB broth (Sigma) supplemented with 100 μg/ml ampicillin as the selection agent. At an optical density of OD_600_ = 0.6, the bacterial cells were induced with 0.5 mM IPTG and, after an additional 3 h of culture, harvested by centrifugation at 2880 x g and 4°C for 20 min. To obtain highly purified preparations of the recombinant proteins, the collected bacterial cells were lysed using the BugBuster Protein Extraction Reagent (Novagen) supplemented with 1 mg/ml lysozyme (Sigma), 25 U/ml benzonase nuclease (Sigma) and 10 μg/ml phenylmethanesulfonyl (PMSF) (Sigma) following the manufacturer’s recommendations. Because the preliminary experiments revealed an overwhelming abundance of the recombinant proteins in the insoluble fraction of inclusion bodies, all of these proteins were isolated under denaturing conditions by immobilized metal affinity chromatography (IMAC) with HisPur^TM^ Cobalt Spin Columns (Thermo Scientific Pierce) according to the manufacturer’s procedure. Briefly, the purified inclusion bodies were first dissolved in Binding Buffer (Novagen) supplemented with 6 M urea (Sigma) and 10 μg/ml PMSF. To remove the insoluble debris, the obtained protein extract was centrifuged at 16,000 x g and 4°C for 30 min, and the supernatant was then mixed with an equal volume of Equilibration/Wash buffer at pH 7.4 [50 mM Na_3_PO_4_ (Sigma), 300 mM NaCl (Sigma), 6 M urea, and 10 mM imidazole (Sigma)] and applied to a column with an equilibrated Co^2+^-charged affinity chromatography resin. The purified recombinant rAtsG, rSerA, rSahH or rGlmU protein was eluted from the resin by the addition of Elution Buffer at pH 7.4 (50 mM Na_3_PO_4_, 300 mM NaCl, 6 M urea, and 150 mM imidazole) and finally analyzed by SDS-PAGE electrophoresis with a 12% polyacrylamide gel and staining with Imperial Protein Stain (Thermo Scientific Pierce). The concentration of each purified recombinant protein in the collected eluates was quantified by the Bradford method using the Bradford Reagent. Furthermore, the recombinant rAtsG, rSerA, rSahH and rGlmU were identified using the Western blotting technique. The electrophoretically separated proteins were transferred to a nitrocellulose membrane for immunological detection with anti-His Tag mouse monoclonal antibodies (Novagen) and horseradish peroxidase (HRP)-conjugated anti-mouse IgG goat polyclonal antibodies (Jackson ImmunoResearch), which were used as primary and secondary antibodies, respectively. The working concentrations of both antibodies were optimized experimentally to 0.2 μg/ml (primary antibodies) and 0.4 μg/ml (secondary antibodies). Finally, the developed immune complexes were visualized using 4-chloro-1-naphtol (Sigma) as the chromogen, and the recombinant protein bands were evaluated based on the theoretically calculated molecular masses.

### Development of mouse polyvalent sera

Female, 8- to 12-week-old BALB/c mice were subcutaneously immunized with three doses (150 μg, 100 μg and 100 μg) of rAtsG, rSahH, rGlmU or rSerA *Mtb* protein, which were administered at two-week intervals. Ten days after the last booster, blood was collected from the immunized animals, and the sera were prepared, aliquoted and stored at -20°C. The titers of the antigen-specific polyclonal IgG antibodies (primary antibody) and optimal experimental dilutions of the developed mouse immune sera were estimated with indirect immunoenzymatic ELISA assay using recombinant rAtsG, rSahH, rGlmU or rSerA protein as the antigen and HRP-conjugated anti-mouse IgG goat polyclonal immunoglobulins as the secondary antibody. The immunoenzymatic reaction was revealed with ABTS (2,2’-azino-bis(3-ethylbenzothiazoline-6-sulfonic acid) diammonium salt) (Sigma) as the chromogen at a concentration of 1 mg/ml in phosphate-citrate buffer at pH 4.5. After 20 min of incubation of the tested samples in the dark, the absorbance values were measured at λ = 405 nm using a MultiScan ELISA reader (ThermoScientific).

The laboratory BALB/c mice used for the experiments were raised under standard conventional conditions in the approved by Polish Ministry of Science and Higher Education animal facility of the Institute of Microbiology, Biotechnology and Immunology, Faculty of Biology and Environmental Protection, University of Lodz. The applied procedures were approved and conducted according to guidelines provided by the appropriate Polish Local Ethics Commission for Experiments on Animals No. 9 in Lodz (Agreement 54/ŁD1/2011).

### Binding of human IL-8 by rAtsG, rSerA, rSahH and rGlmU

To confirm the binding of the affinity chromatography-selected *Mtb* proteins to IL-8, the Western blotting procedure with recombinant rAtsG, rSerA, rSahH and rGlmU was employed. The mycobacterial proteins were separated by SDS-PAGE electrophoresis on a 12% polyacrylamide gel and then transferred to a nitrocellulose membrane. After overnight blocking with WesternDot^TM^ blocking buffer (Invitrogen) at 4°C, the immobilized rAtsG, rSerA, rSahH and rGlmU were incubated with recombinant human IL-8 (PROSPEC) at a concentration of 5 μg/ml for 2 h at room temperature. The membrane was then washed with PBS/0.05% Tween20, and the bound human chemokine was detected with anti-human IL-8 goat polyclonal IgG (R&D Systems) at a concentration of 1 μg/ml in PBS/0.05% Tween20 at room temperature for 1 h. The immunoenzymatic reaction was developed with HRP-conjugated rabbit anti-goat IgG (0.4 μg/ml) as the secondary antibody and the 3,3′,5,5′-Tetramethylbenzidine (TMB) Liquid Substrate System for Membranes (Sigma) as the chromogen.

### Expression and purification of soluble (native) forms of recombinant rAtsG, rSahH and rGlmU proteins

To prepare native (soluble) forms of rAtsG, rSahH and rGlmU proteins of *Mtb*, a recombinant strain of *E*. *coli* BL12(DE3) transformed with the pHis-atsG, pHis-sahH or pHis-glmU plasmid was grown at 37°C in LB broth supplemented with 100 μg/ml ampicillin until the optical density of the bacterial culture reached OD_600_ = 0.6. After cooling to room temperature, the bacterial cells were induced to overproduce the recombinant proteins through a 4-h incubation with IPTG at a final concentration of 0.4 mM at 25°C with constant shaking. The *E*. *coli* cells were collected by centrifugation at 8000 x g and 4°C for 10 min, resuspended in 5 ml of 1X Binding Buffer with 1 mM PMSF, 100 μg/ml lysozyme, and 10 U/ml benzonase nuclease and then incubated for 30 min on ice. Finally, the bacterial cells were disrupted using a Bioblock Scientific Ultrasonic Homogenizer equipped with a 3-mm sonotrode (Labo Plus) by applying 10 cycles of sonication for 10 s at 1-min intervals. After sonication, the bacterial cellular debris was removed by centrifugation at 13,000 x g and 4°C for 40 min. The supernatants containing the soluble recombinant mycobacterial proteins of interest were filtered through nitrocellulose filters with a pore size of 0.45 μm and then subjected to purification using a His Bind Purification Kit (Novagen) according to the manufacturer’s indications with slight modification. Briefly, each supernatant was passed five times through the column with His Bind affinity resin, and after washing, the recombinant protein was eluted with 10 ml of 1X Elution Buffer. The presence of soluble rAtsG, rSahH and rGlmU proteins in the collected 1-ml fractions was analyzed by SDS-PAGE electrophoresis on a 12% polyacrylamide gel and staining with Imperial Protein Stain. To exchange the Elution Buffer with imidazole in TRIS/NaCl buffer at pH 7.5, the eluates of the recombinant proteins were applied into PD 10 Columns-CGE (Healthcare) and then concentrated using 30-kDa Amicon Ultra Centrifugal Filter Units (Millipore) to a final volume of 1 ml. To preserve protein stability, glycerol was added to each sample to a final concentration of 10% (v/v), and the solutions of rAtsG, rSahH and rGlmU were aliquoted and stored at -80°C.

### Surface Plasmon Resonance

To confirm the affinity chromatography results, the interaction of human IL-8 with recombinant mycobacterial rAtsG, rSahH and rGlmU was further evaluated through Surface Plasmon Resonance (SPR). Human recombinant IL-8 chemokine (Life Technologies) was immobilized on a CM5 sensor chip (flow cell 2- Fc2) by the amine coupling method (Biacore AB, Uppsala, Sweden) according to the manufacturer's instructions. Additionally, as a control to each experimental set, BSA was immobilized on a CM5 sensor chip in the same manner (flow cell 1—Fc1). The experiments were performed at 22°C in PBS buffer (pH 7.4) containing the surfactant P20 at a concentration of 0.005% (v/v). Before the analysis, the buffer of the soluble rAtsG, rSahH and rGlmU proteins was changed to PBS with the PD 10 columns, and the proteins were then diluted with the latter buffer to obtain the same range of concentrations, namely 2.5 μM, 5 μM, 10 μM, and 20 μM, which were used in 30-μl aliquots for injection. Complex formation was observed at a flow rate of 5 μl/min. Kinetic data were calculated and statistically analyzed using the BIAevaluation 3.2 software supplied by Biacore AB, with at least three independent runs of each concentration and Chi^2^ test to verify mathematical model of interaction. The results are expressed in resonance units (RU), an arbitrary unit specific for the Biacore instrument (1000 RU corresponds to ∼1 ng of bound protein/mm^2^), as a result of Fc2-Fc1. The association (k_a_) and dissociation (k_d_) rate constants were determined from individual association and dissociation phases, respectively, assuming one-to-one interactions.

### Mycobacterial overproduction constructs

The overexpression vectors were engineered by amplifying the *atsG*, *glmU*, *and sahH* genes using *Mtb* chromosomal DNA as a template. The PCR products flanked by appropriate restriction enzyme recognition sites (Table 1) were initially cloned into pJet1.2, verified by sequencing and subsequently re-cloned into the pKW08 episomal vector under the control of the *P*_*tet*_ promoter using EcoRI and HindIII (*atsG*) or BamH and HindIII (*glmU* and *sahH*). The resultant *Mtb* mutant strains overproducing AtsG, GlmU or SahH protein were denoted *Mtb*AtsG↑, *Mtb*GlmU↑, and *Mtb*SahH↑, respectively.

### Analysis of *Mtb* mutant strains

The Western blotting technique was used to evaluate the overproduction of AtsG, GlmU and SahH proteins by the constructed *Mtb*AtsG↑, *Mtb*GlmU↑, and *Mtb*SahH↑ mutants. Mycobacteria harvested from 100 ml of the suspension culture of the mutant and”wild-type” *Mtb* (control cells) strains were subjected to preparation of whole-cell lysates enriched in the membrane protein fractions, as mentioned above. After determination of protein concentrations, 25 μg of the protein extracts was electrophoretically separated on 12% polyacrylamide gels by SDS-PAGE and then transferred to a nitrocellulose membrane for further immune detection. The proteins of interest were identified with the respective antigen-specific mouse polyvalent sera (primary antibody), diluted 1:500 in 1% skim milk in PBS, and HRP-conjugated anti-mouse IgG goat polyclonal immunoglobulins as a secondary antibody (0.4 μg/ml). The developed immune complexes were visualized using the 3,3′,5,5′-Tetramethylbenzidine (TMB) Liquid Substrate System for Membranes as the chromogen and analyzed based on their molecular masses.

The optimal dilutions of the specific mouse immunoglobulins were determined in preliminary titration experiments.

### Neutrophil infection and mycobacterial attachment/entry determination

Blood was drawn from healthy volunteers, and neutrophils were isolated according to the protocol routinely used in our laboratory [36]. The purified neutrophils, which were maintained in RPMI 1640 medium supplemented with 2 mM L-glutamic acid, 1 mM sodium pyruvate and 10% fetal bovine serum (Sigma), were infected with “wild-type” *Mtb*, *Mtb*AtsG↑, *Mtb*GlmU↑ or *Mtb*SahH↑ strain (MOI of 1: 10) for 30 min at 37°C with 5% CO_2_. Extracellularly located (unbound) mycobacteria were then washed off. The infected phagocytes were lysed with 1mL of 0.1% sodium dodecyl sulfate (SDS) (Sigma) in PBS, and appropriate dilutions of the cell lysates were plated onto Middlebrook 7H10 agar supplemented with 10% OADC enrichment. After 14 days of culture, the CFUs were counted. In some experiments, “wild-type” *Mtb*, *Mtb*AtsG↑, *Mtb*GlmU↑, and *Mtb*SahH↑ tubercle bacilli were incubated with recombinant human IL-8 overnight at 4°C, washed with PBS, and then used for infection. The final concentration of the human chemokine used in these experiments was 100 ng/ml per 5×10^7^ *Mtb* cells.

To estimate the effect of the AtsG, GlmU and SahH overproduction as well as IL-8 opsonization on the mycobacterial attachment/entry into human neutrophils, two independent sets of experiments were performed. The experiments were run in nine (first set) or five (second set) repetitions.

### Statistical analysis

Data represented by means ± SD were analyzed using the Mann-Whitney U test. In some cases, statistical evaluation of data for more than two experimental groups was performed with One-Way ANOVA. *P* values < 0.05 were regarded as statistically significant.

## Results

### Purification of *Mtb* proteins engaged in human IL-8 binding

**Fig 1:**
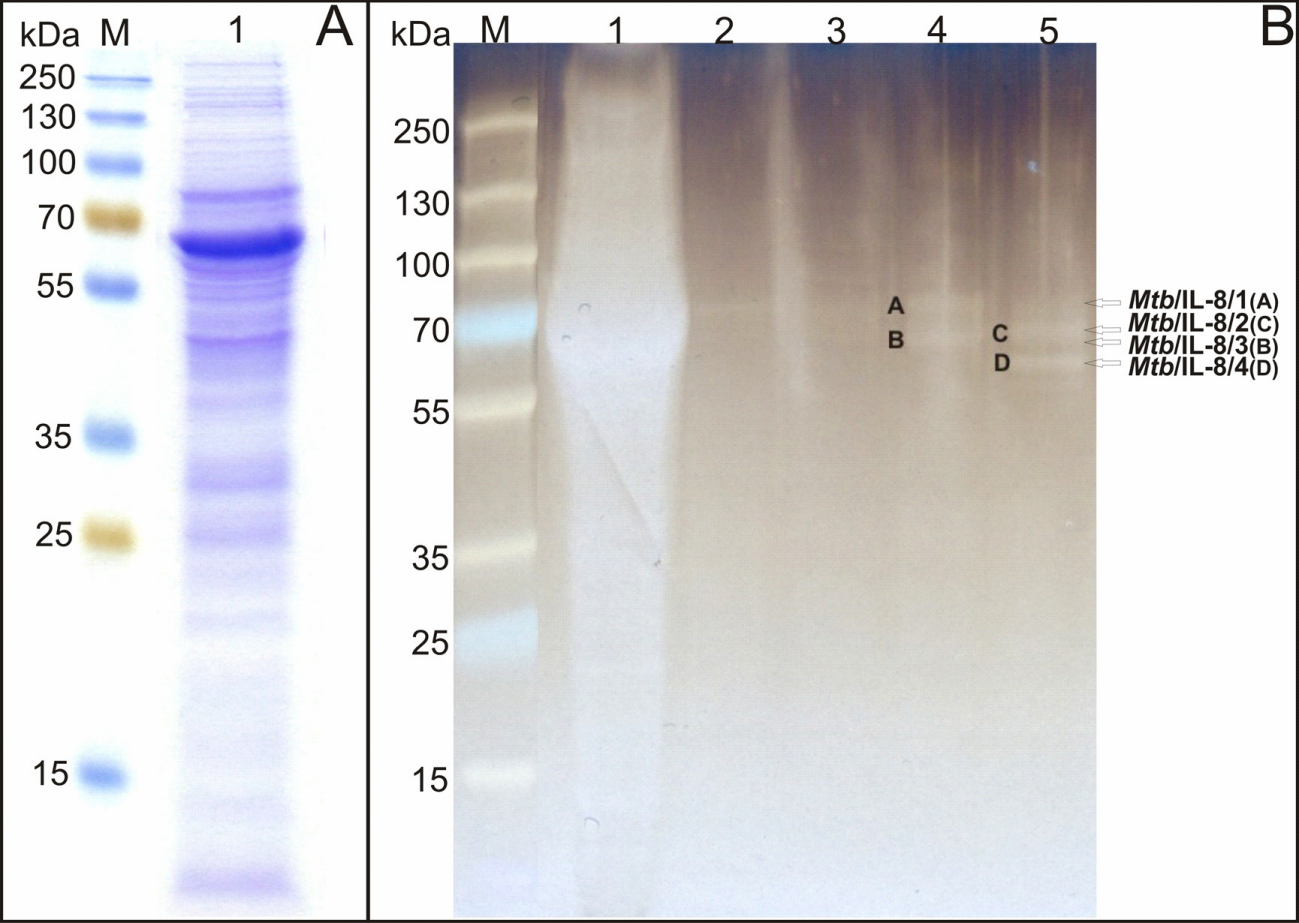
SDS-PAGE analysis of the affinity chromatography-purified *M*. *tuberculosis* proteins interacting with human IL-8. (A) Mycobacterial whole-cell extract applied onto the agarose resin (lane 1). (B) Affinity chromatography-purified *Mtb* proteins binding human IL-8: lane 1-mycobacterial whole-cell lysate, lane 2-flowthrough fraction collected after the washing step, lane 3-eluate fraction 1, lane 4-eluate fraction 2, lane 5-eluate fraction 3. M-protein molecular weight standard.

Employment of the methodology for the preparation of broken mycobacterial cell extract [32] allowed development of *Mtb* whole-cell lysates containing a diverse spectrum of the mycobacterial membrane and cytoplasmic proteins (Fig 1A). The concentration of the extracted proteins differed between the preparations and ranged from 0.995 to 1.79 mg/ml, corresponding to a total protein yield ranging from 4.975 to 8.95 mg per 100 ml of the bacterial culture, respectively.

To separate human IL-8-binding *Mtb* cell component/components, the extracted mycobacterial proteins were then subjected to protein-protein interactions using the affinity chromatography technique with a human chemokine coupled-beaded agarose resin via the reaction of primary amines and functional aldehyde groups, respectively. The SDS-PAGE profile of the eluates obtained by affinity chromatography revealed four intense protein bands of approximate molecular masses ranging from 60 to 80 kDa (Fig 1B). These proteins were mainly detected in the eluate fractions 2 and 3 and were designated as the following human IL-8-binding *Mtb* components: *Mtb*/IL-8/1 (band A; ~75–80 kDa), *Mtb*/IL-8/2 (band B; ~65–70 kDa), *Mtb*/IL-8/3 (band C; ~70 kDa), and *Mtb*/IL-8/4 (band D; ~60 kDa).

### Identification of human IL-8-binding *Mtb* proteins

The affinity chromatography-selected *Mtb* proteins that presumably bind to the human IL-8 chemokine were further identified with the LC/MS/MS analytical tool. Identification based on LC/MS/MS sequencing of at least two independent peptides (a confidence level of at least 99%) from the same analyzed protein was required to include the candidate protein into the list of potential IL-8 ligands. As a final result, four candidate mycobacterial proteins, namely AtsG (Rv0296c; arylsulfatase), GlmU (Rv1018c; bifunctional glucosamine-1-phosphate acetyltransferase and N-acetylglucosamine-1-phosphate uridyltransferase), SahH (Rv3248c; S-adenosyl-L-homocysteine hydrolase) and SerA (Rv2996c; D-3-phosphoglycerate dehydrogenase SerA1) were selected and subjected to further analysis. Proteins and peptides identification details are provided in S1 Table in supplementary materials related to this article.

### Analysis of *Mtb* AtsG, GlmU, SahH and SerA protein interactions with human IL-8 chemokine

**Fig 2:**
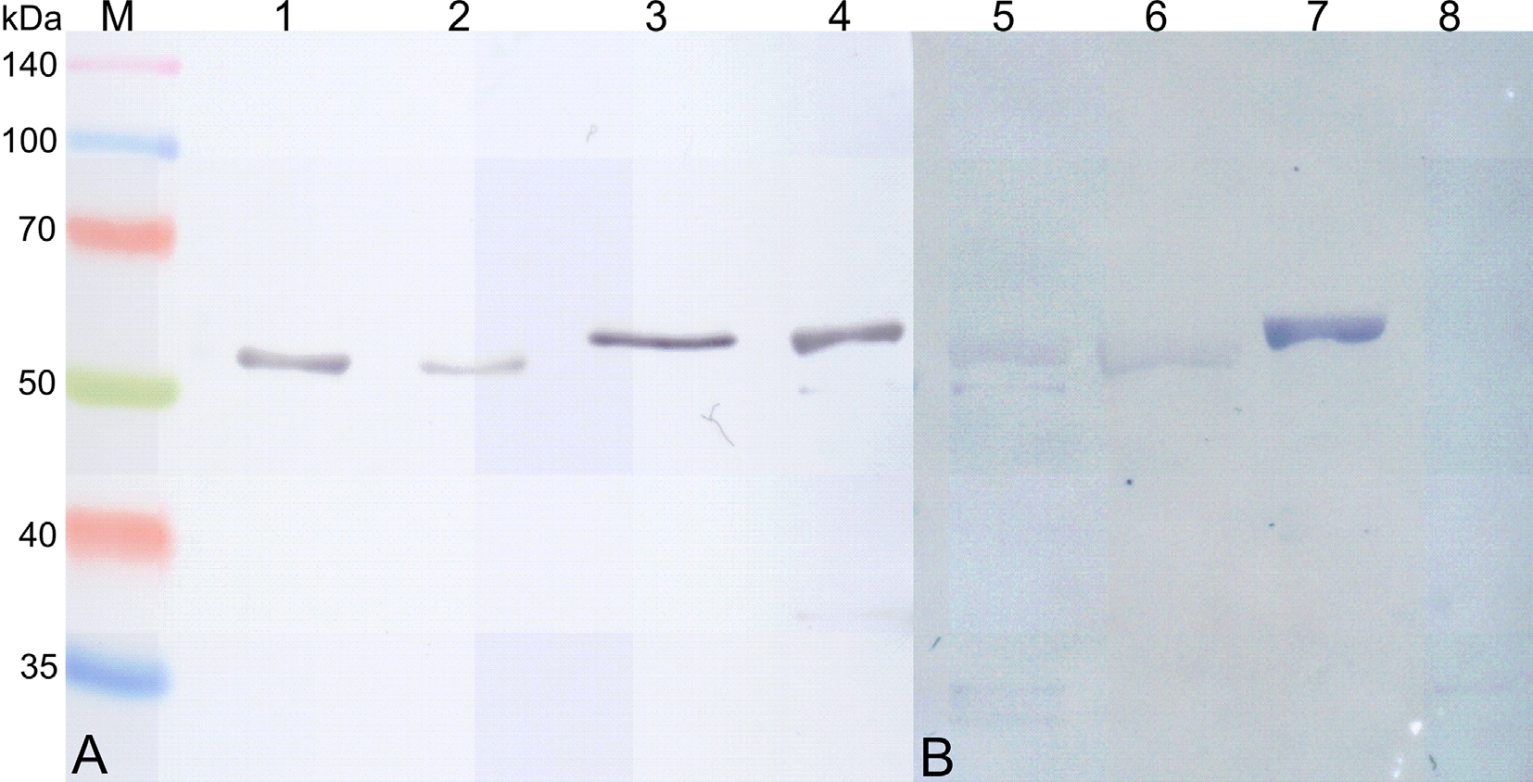
Western blotting analysis of recombinant *M*. *tuberculosis* rAtsG, rGlmU, rSahH, rSerA proteins developed in *E*. *coli* and their interactions with human IL-8. (A) Immunodetection of recombinant *Mtb* rAtsG (lane 1), rGlmU (lane 2), rSahH (lane 3) and rSerA (lane 4) with anti-His Tag mouse monoclonal IgG1 antibodies. (B) Human IL-8 binding by recombinant *Mtb* rAtsG (lane 5), rGlmU (lane 6), rSahH (lane 7) and rSerA (lane 8). M-protein molecular weight standard.

To analyze in detail and confirm the interactions of the LC/MS/MS-identified mycobacterial proteins with human IL-8, the recombinant rAtsG, rGlmU, rSahH and rSerA proteins were employed. The SDS-PAGE analysis of rAtsG, rGlmU, rSahH and rSerA revealed that the expressed recombinant proteins were successfully purified by immobilized metal affinity chromatography and migrated with molecular masses of 55.2, 55.1, 57.9, and 58.1 kDa, respectively, consistent with those calculated based on the amino acid sequences. The accurate purity of the recombinant protein preparations was estimated by densitometry using the FluorChem 8800 software (Alpha Innotech Corp.) and was greater than 95%. Additionally, identification of the rAtsG, rGlmU, rSahH and rSerA proteins with the Western blotting technique confirmed the previous results obtained by SDS-PAGE analysis (Fig 2A). The concentrations of rAtsG, rGlmU, rSahH and rSerA in the eluted samples ranged from 0.1 to 2.8 mg/ml depending on the affinity chromatography eluate fraction, but the calculated total protein yields were 128 mg, 52 mg, 59 mg and 74 mg, respectively, in 1 L of the IPTG-induced bacterial culture.

To assess their ability to interact with a human IL-8, the recombinant mycobacterial ligands were subjected to chemokine binding using the Western blotting technique. The performed experiment showed that, of the four affinity chromatography-pre-selected mycobacterial ligands for human IL-8, only three proteins, namely rAtsG, rGlmU and rSahH, bound the chemokine (Fig 2B). A protein-cytokine interaction was not confirmed for the *Mtb* rSerA protein.

**Fig 3:**
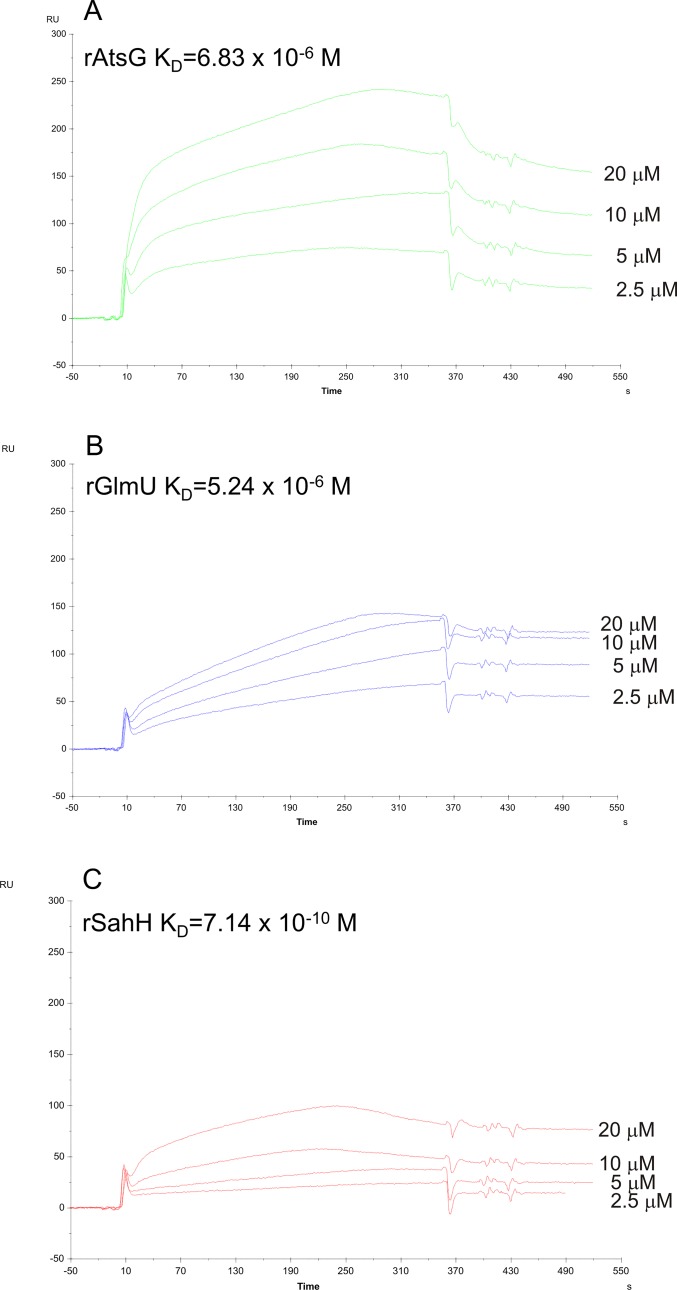
Surface plasmon resonance evaluation of protein-protein interplay between recombinant *M*. *tuberculosis* rAtsG, rGlmU, rSahH proteins and human IL-8. Sensorgrams of the binding interaction between IL-8 immobilized onto the CM5 sensor chip and (A) *Mtb* rAtsG, (B) *Mtb* rGlmU or (C) *Mtb* rSahH protein at final concentrations of 2.5 μg, 5 μg, 10 μg and 20 μg at an analyte flow rate of 5 μl/min. The presented kinetic data were calculated from at least three independent runs of each concentration.

A final evaluation of the binding of human IL-8 to mycobacterial AtsG, GlmU or SahH protein was accomplished using the Surface Plasmon Resonance (SPR) analytical methodology. To identify the molecular interaction between human IL-8 and *Mtb* AtsG, GlmU or SahH protein by SPR, the chemokine served as a target partner (ligand molecule) and was attached to the surface of a sensor surface. The recombinant soluble (native) forms of the mycobacterial proteins were used as the second partner (analyte) of the examined interaction and were passed over the surface with the immobilized ligand in a continuous flow of the sample solution in the separate experiments. As a result of differences in the refractive index between the running buffer and the injected sample, the bulk shift in the SPR response was evaluated, and the k_a_ and k_d_ kinetic parameters were determined using the Biacore BIAevaluation 3.2 software. Fig 3 represents the SPR sensorgrams picturing the kinetics of the mycobacterial rAtsG (A), rGlmU (B) or rSahH (C) interactions with human IL-8. The highest binding affinity was detected for the IL-8-rSahH interaction, which had a K_D_ of 7.14x10^-10^ M. Moreover, a significant, albeit less strong, protein-protein interaction to the human chemokine was also found for rAtsG and rGlmU, with K_D_ values of 6.83x10^-6^ M and 5.24x10^-6^ M, respectively.

### Overproduction of AtsG, GlmU and SahH proteins by *Mtb* intensifies bacterial attachment/entry into human neutrophils

**Fig 4:**
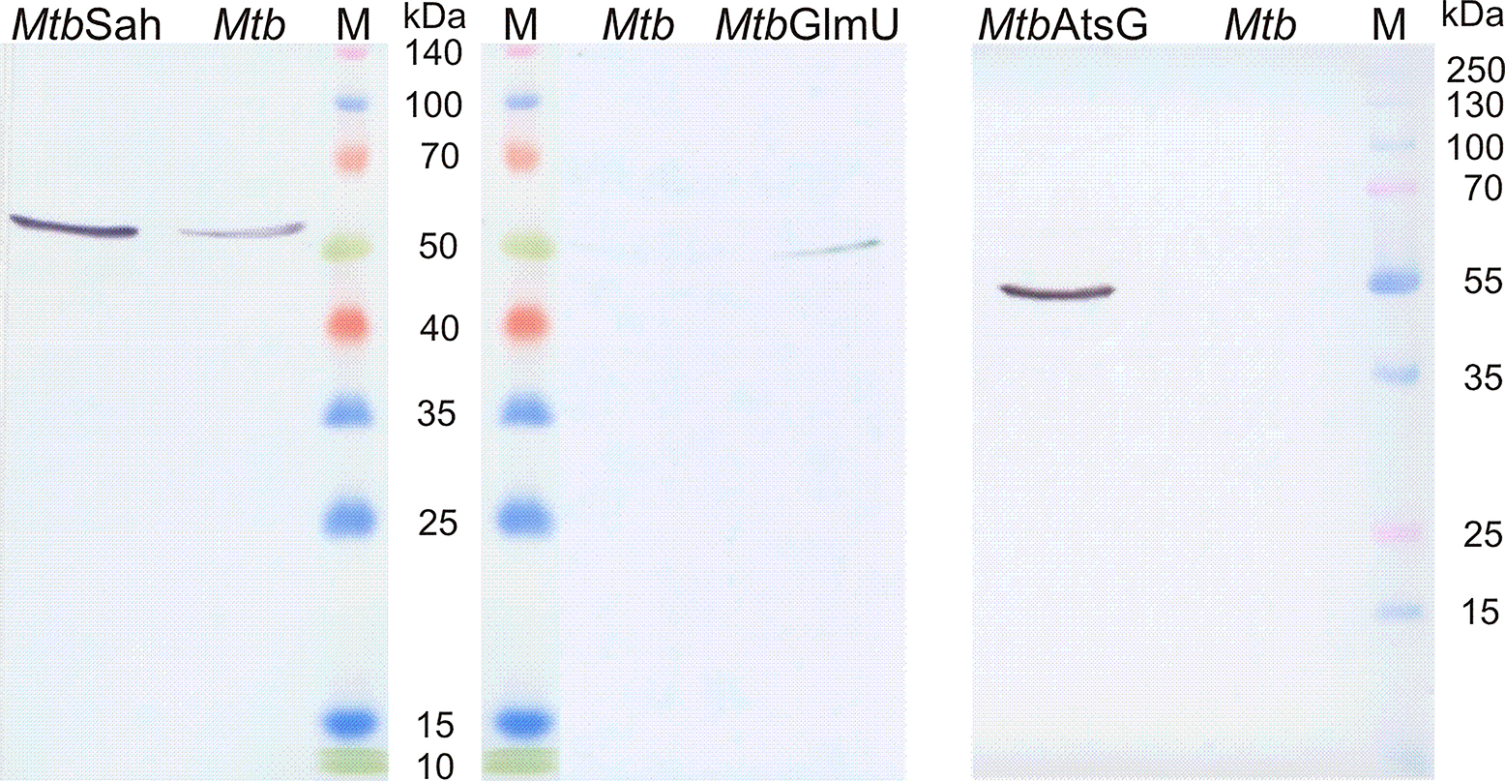
Analysis of *M*. *tuberculosis* overproducing mutants. Western blotting determination of the level of AtsG, GlmU or SahH protein biosynthesis by *Mtb*AtsG↑, *Mtb*GlmU↑ or *Mtb*SahH↑ overproducing mutant strain, respectively, compared with the “wild-type” *Mtb* strain. M-protein molecular weight standard.

To evaluate the significance of the human IL-8-binding *Mtb* proteins during the course of the mycobacterial infection of host cells, *Mtb* mutants overproducing the AtsG, SahH or GlmU antigen were constructed. The resultant merodiploid strains were analyzed with respect to AtsG, SahH or GlmU protein biosynthesis and the levels of protein overproduction by the mutant strains were compared to their levels synthetized by the “wild-type” *Mtb* strain. The source of the mycobacterial AtsG, GlmU or SahH protein subjected to the comparative study was a whole-cell extract prepared from the respective bacterial strain. The whole protein concentration in the extracts reached 1.35 mg/ml, 1.41 mg/ml and 1.21 mg/ml in the preparations of *Mtb*AtsG↑, *Mtb*GlmU↑ and *Mtb*SahH↑ cells, respectively. An analysis of the protein bands developed by an immunoenzymatic reaction revealed that all of the constructed *Mtb* strains overproducing AtsG, GlmU or SahH were characterized by an increased synthesis of the respective target antigen compared with the levels of the proteins produced by the cells of “wild-type” *Mtb* (Fig 4). The highest antigen overproduction was detected for the *Mtb*AtsG↑ strain compared with the “wild-type” *Mtb* as well as the other constructs. Additionally, we also noted relatively low level of GlmU protein synthesis compared with the production levels of AtsG and SahH. The comparative analysis of the *Mtb*GlmU↑ and “wild-type” *Mtb* strains required application of 2 times greater amount of whole protein, namely 50 μg/well instead of 25 μg/well in the case of AtsG and SahH, for both antigen detection and estimation of differences in GlmU synthesis.

**Fig 5:**
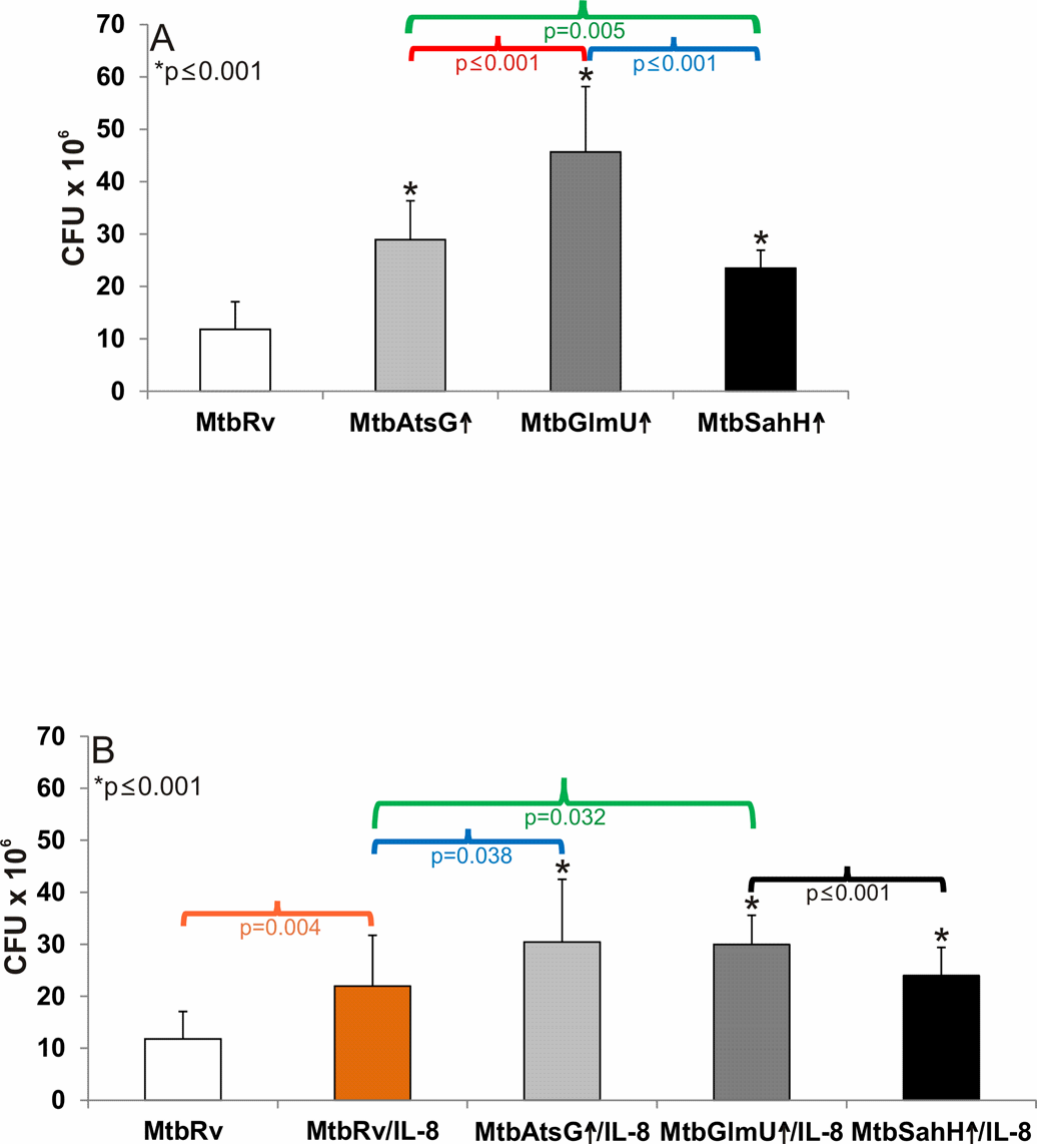
Overproduction of AtsG, GlmU or SahH protein increases the attachment/entry of *M*. *tuberculosis* into human neutrophils. (A) Evaluation of the engulfment of the *Mtb* mutant strain overproducing AtsG (*Mtb*AtsG↑), GlmU (*Mtb*GlmU↑) or SahH (*Mtb*SahH↑) protein by human neutrophils compared with that of the “wild-type” *Mtb* strain. (B) Effect of prior exposure to IL-8 on the attachment/entry of *Mtb*AtsG↑, *Mtb*GlmU↑, *Mtb*SahH↑ overproducing mutants into human neutrophils compared with IL-8-pre-incubated and IL-8-untreated “wild-type” *Mtb* strain. Data are presented as mean values (±SD) from two independent experiments comprising of nine or five repetitions, respectively.

To estimate the effect of AtsG, GlmU and SahH overproduction on early events of *Mtb* infection, the constructed *Mtb*AtsG↑, *Mtb*GlmU↑ and *Mtb*SahH↑ strains as well as the “wild-type” *Mtb* were further used in phagocytosis experiments with human neutrophils, which are important leukocytes involved in the elimination of the pathogen from the host. As shown in Fig 5A, overproduction of the mycobacterial AtsG (p≤0.001), GlmU (p≤0.001) or SahH (p≤0.001) protein resulted in a significant augmentation of pathogen interaction (attachment/entry) with human neutrophils compared with the control “wild-type” strain. The highest statistically significant number of attached/intracellularly located mycobacterial cells, namely 4.6±1.3x10^7^, was found in neutrophil cultures infected with the *Mtb* strain overproducing the GlmU antigen compared with the number of “wild-type” *Mtb* (1.2±0.5x10^7^) as well as the *Mtb*AtsG↑ (2.9±0.7x10^7^; p≤0.001) and *Mtb*SahH↑ (2.3±0.3x10^7^; p≤0.001) bacilli interacting with human neutrophils. Moreover, a statistical analysis of the data obtained for *Mtb*AtsG↑ and *Mtb*SahH↑ phagocytosis also appeared to show that the observed diversity in the number of attached/engulfed mycobacteria was statistically significant (p = 0.005).

### Interleukin-8-opsonization enhances attachment/entry of “wild-type” *Mtb*, but not AtsG, GlmU and SahH overproducing mutant strains, into human neutrophils

Because the above-presented data clearly indicate the involvement of the mycobacterial AtsG, GlmU and SahH proteins in the interactions with human IL-8, which is considered a crucial chemokine of the immune response to *Mtb* infection, it appeared to be extremely valuable to determine the importance of this phenomenon in the course of bacterial cell engulfment. To estimate the supposed engagement of the IL-8 chemokine in the opsonization stage of mycobacterial phagocytosis, “wild-type” *Mtb* as well as the mutant strains overproducing AtsG, GlmU or SahH protein were exposed to the chemokine and then were used to infect human neutrophils (MOI of 10 bacilli/1 host cell). Prior exposure to IL-8 (Fig 5B) resulted in a significant (p = 0.004) almost two-fold increase in the attachment/uptake of “wild-type” *Mtb* (2.2±0.9x10^7^) by human neutrophils compared with the control bacteria uncoated with the chemokine (1.2±0.5x10^7^). Although overproduction of AtsG, GlmU and SahH significantly intensified interactions of mutant strains with human phagocytes compared with “wild-type” *Mtb*, pre-treatment with IL-8 did not improve the phagocytosis of *Mtb*AtsG↑ (3±1.2x10^7^), *Mtb*GlmU↑ (3±0.6x10^7^) and *Mtb*SahH↑ (2.4±0.3x10^7^) mutants compared with the respective IL-8-non-opsonized overproducing strain (Fig 5B). Therefore the enhanced attachment/entry of IL-8-coupled AtsG- (p = 0.038), GlmU- (p = 0.032) and SahH-overproducing mycobacteria into human neutrophils compared with IL-8-opsonized “wild-type” *Mtb* was due to the augmented protein expression but not IL-8 binding. Additionally, in the case of GlmU-overproducing bacilli, prior exposure to the chemokine led to an unexpected decrease in the number of attached/intracellularly located mycobacteria. Moreover, although there were no differences between the phagocytosis of IL-8-coated *Mtb*AtsG↑ and *Mtb*GlmU↑ or *Mtb*SahH↑ mycobacteria, a significantly higher number of tubercle bacilli overproducing the GlmU protein were uptaken compared with that of *Mtb*SahH↑ (p = 0.008).

## Discussion

*Mycobacterium tuberculosis* is a highly successful intracellular pathogen armed with a plethora of mechanisms that ensure its adaptation to and replication in the hostile environment inside phagocytes. The mycobacterial pathogenic strategies rely on selective interactions of its cell wall and secretory effectors with host targets involved in the development of an effective anti-microbial immunity. These interactions lead to the manipulation and disturb the expansion of the host innate and adaptive immune responses, respectively. The resulting effect of such relationships is an achievement of the optimal conditions for intracellular survival of the pathogen. We recently reported the binding interaction of virulent *Mtb* with the human IL-8 chemotactic cytokine [28]. Additionally, competitive inhibition experiments revealed that the observed host chemokine-pathogen interaction was specific and positively influenced *Mtb* phagocytosis at both stages, namely engulfment and intracellular killing.

In the present manuscript, we document the existence of mycobacterial molecular effectors responsible for selective binding to human IL-8. The application of affinity chromatography, LC/MS/MS and SPR techniques allowed identification of tubercle bacilli AstG (Rv0296c), GlmU (Rv1018c) and SahH (Rv3248c) proteins as IL-8-binding ligands. Currently, the involvement of these mycobacterial constituents in the pathogenesis of tuberculosis is unknown, and none of these have been previously reported to interact with the host target substrates or molecular pathways. However, all of these proteins have been implicated to determine *Mtb* virulence. Interaction of AtsG, GlmU and SahH proteins with both human IL-8 and host cell components is extremely intriguing in the context of tubercle bacilli pathogenicity, because all identified herein *Mtb* IL-8-effectors are constituents of membrane protein fraction of pathogen. Additionally, AtsG and SahH are also detected in a culture filtrate as well as in a whole cell lysate of mycobacteria [37].

Identified by Hossian et al. [38] the *Mtb* AtsG arylsulfatase is a member of the sulfatase family of enzymes that catalyze the hydrolysis of sulfate esters (or N-sulfates) to an alcohol (or amine) and free sulfate [39]. Together with sulfotransferases, which are responsible for the biosynthesis of sulfate compounds, sulfatases are essential elements of a complex mycobacterial sulfur metabolism that have been linked to the virulence of this pathogen. Because sulfated molecules are mediators of cell-cell interactions, the sulfur machinery of *Mtb* is considered to regulate host-pathogen interactions as well as to facilitate the metabolic adaptation of the pathogen to the hostile host environment. Although the biosynthesis of sulfur-containing compounds (e.g., cell wall-associated sulfolipid-1, cysteine, methionine, and mycothiol) determines mycobacterial virulence, detoxification pathways and interactions with the host immune mechanisms, the desulfation of biomolecules, which is catalyzed by sulfatases, could also impact diverse biological processes and may be critical for a number of processes, e.g., pathogen adhesion and nutrient scavenging [39,40]. In our studies, we found that the overexpression of AtsG arylsulfatase contributes to a significant increase in the number of *Mtb* cells attached to/engulfed by human neutrophils. This observation supports the previous suggestion regarding the engagement of mycobacterial sulfatases in the bacterial adhesion phenomenon [39]. It was indicated that mycobacterial sulfatases may participate in the desulfation of host extracellular glycosaminoglicans. A consequence of the remodeling of sulfoforms in the extracellular matrix may be modulation of bacterial cell adhesion, resulting in augmentation of intracellularly located mycobacteria. Furthermore, the interaction of sulfatases with cell surface or cell surface-attached sulfated glycosaminoglycans could also occur. The attachment of *Mtb* to epithelial cells and the extracellular matrix was previously proposed to depend on interactions with sulfated glycoconjugates [41]. Additionally, the correlation between the activity of sulfatases and pathogenicity has been described for many microorganisms, including lung pathogens [42,43].

The interesting aspect of *Mtb* AtsG arylsulfatase biological activity is its interaction with the IL-8 chemokine. The consequence of such interplay between the pathogen and host proteins could be an expansion of the repertoire of host ligands utilized by mycobacteria to gain an intracellular location. It needs to be highlighted, however, that the contribution of AtsG/IL-8 interaction to tubercle bacilli entry into host cells is still a controversial issue. The SPR analysis revealed that the binding of human IL-8 to *Mtb* AtsG arylsulfatase is characterized by a high binding affinity (K_D_ = 6.83x10^-6^ M) similar to that noted for the interaction of IL-8 with sulfated glycosaminoglycans, namely chondroitin-6-sulfate (K_D_ = 1.4±0.4x10^-6^ M) [44] and heparin (K_D_ = 2.0±0.4x10^-6^ M) [45]. However, the binding constants for IL-8 and its chemokine receptors are considerably higher (K_D_ = 1-7x10^-9^ M) [46,47]. Surprisingly, in contrast to the results obtained for the “wild-type” *Mtb*, prior exposure of the AtsG-overproducing *Mtb* strain to IL-8 did not result in further magnification of pathogen uptake by human phagocytes compared with the untreated respective mutant. This result may be due to an insufficient IL-8 concentration used for opsonization of the mutant cells because the optimal amount of the chemokine (final concentration of 100 ng/ml per 5×10^7^ tubercle bacilli) used for the pre-treatment of “wild-type” mycobacteria could be unsaturable in the case of the AtsG-overproducing strain. However, a detailed study is necessary to fully clarify the molecular mechanisms underlying the interplay between AtsG and IL-8, including determination of the protein regions involved in this binding.

As mentioned above, the interaction of AtsG arylsulfatase with human IL-8 complexed with sulfated glycosaminoglycans could also participate in essential nutrient scavenging. Mycobacterial sulfatases constitute the necessary pathogen tools that facilitate the acquisition of sulfate from the host environment or from endogenous metabolites [39,40]. Additionally, bacterial sulfatases may be involved in the breakdown of sulfated molecules for use as a source of carbon [48]. Sulfate scavenging is required for the synthesis of the sulfur-containing molecules that determine the invading properties of mycobacteria. Sogi et al. recently described the role of *Mtb* type II alkylsulfatase Rv3406 in sulfate uptake with 2-ethylhexyl sulfate as the sole sulfur source [48]. The participation of AtsG arylsulfatase in sulfate acquisition has never been revealed. The determination of this presumed feature of AtsG as well as the contribution of the AtsG-IL-8 interaction to this process require extensive studies and may be an urgent issue in the search for novel therapeutic targets for *Mtb*.

In addition to the AtsG arylsulfatase, two other mycobacterial proteins, GlmU and SahH, were identified in our studies as pathogen effectors interacting with human IL-8. A particularly intriguing finding was the interplay between SahH, S-adenosylhomocysteine hydrolase, and the chemokine. The estimated binding affinity of SahH to IL-8 is higher (K_D_ = 7.14x10^-10^ M) than the binding affinity of the chemokine with its specific receptors. This finding suggests that the SahH protein can successfully compete with the CXCR1 and CXCR2 receptors for IL-8 binding. The biological role of this interaction is rather difficult to predict because the molecular regulatory mechanisms underlying SahH activity and the participation of the enzyme in the pathogenesis of tuberculosis remain unknown. SahH is described as a central enzyme of methylation-based processes that catalyzes the reversible hydrolysis of S-L-adenosylhomocysteine (SAH) into free adenosine and L-homocysteine. This protein is responsible for maintenance of the proper intracellular SAH and S-adenosylmethionine (SAM) equilibrium [49,50]. Previous studies have indicated that highly related SAM-dependent methyltransferases are pivotal for *Mtb* virulence by determining the high impermeability of the cell envelope composed of mycolic acids [51,52]. The up-regulation of SahH expression in intraphagosomally growing mycobacteria as well as the maintenance of the functionalization of mycolic acids clearly imply the involvement of SahH hydrolase in mycobacterial pathogenesis [49,53,54]. Based on our data, we propose here, that contribution to the pathogen outer cell wall layer integrity is not the only function of SahH. The significantly higher number of attached/intracellularly located cells of the SahH-overproducing *Mtb* mutant compared with the number of engulfed ‘wild-type” mycobacteria reveals SahH hydrolase as an additional mycobacterial effector engaged in the modulation of pathogen adherence to the target host cells.

Another identified herein *Mtb* component interacting with IL-8 was GlmU protein. The binding affinity of GlmU to IL-8 (K_D_ = 5.24x10^-6^ M) is similar to that calculated for AtsG arylsulfatase. Similarly to SahH hydrolase, mycobacterial GlmU is involved in maintaining the permanence of the pathogen’s cell wall. Zhang et al. [55] characterized the mycobacterial GlmU as a bifunctional enzyme that catalyzes the synthesis of UDP-N-acetyl-D-glucosamine (UDP-GlcNAc), a fundamental precursor of peptidoglycan and the rhamnose-GlcNAc linker region of the mycobacterial cell wall. Furthermore, we postulate that the *Mtb* GlmU protein is involved in modifying the interaction of the pathogen with polymorphonuclear phagocytes infiltrating the infected respiratory tissue. Our studies document that mycobacterial GlmU, together with the AtsG arylsulfatase and SahH hydrolase, presumably intensifies mycobacterial attachment to human neutrophils because the overexpression of this enzyme resulted in an increase in the number of engulfed bacilli. However, as observed for *Mtb*AtsG↑, the pre-incubation of the SahH- and GlmU-overproducing mutants with recombinant IL-8 had no effect on the number of attached/intracellularly located pathogens compared with the respective chemokine-unexposed mutant strain. Moreover, IL-8-opsonization of *Mtb*GlmU↑ tubercle bacilli provided a decreased number of engulfed bacteria compared with that obtained with the untreated GlmU-overproducing strain. However, this result must be analyzed with great caution. Our experiments noted significant divergences in the response of neutrophils depending on the cell donor (data not shown). Primarily, these discrepancies pertain to experiments on the IL-8-opsonized overproducing strains. The donor-specific differences in the response of neutrophils to mycobacterial infection are not unexpected. This phenomenon was also described by other researchers [56,57] and is likely related not only to host-specific factors, e.g. genetically determined predispositions of an individual to combat infection, but also to the adaptation of tubercle bacilli to the specific environmental cues that regulate utilization of the pathogenic strategies providing successful survival inside the infected host.

The interaction of pathogenic mycobacteria with the host cytokine network directing and shaping the immune response was found more than two decades ago. In 1990, interleukin-6 was shown to promote extracellular and intracellular *in vitro* growth of *M*. *avium*. Further analysis revealed that this pathogenic mycobacteria possess a specific receptor for the cytokine (K_D_ = 5x10^-10^ M) with the estimated 15,000 receptor sites per bacterial cell [58,59]. Transforming growth factor β1 [60] and, in an iron-starvation environment, tumor necrosis factor α [61] have a growth-promoting effect on *Mtb* in human monocytes. Bermudez et al. [62] demonstrated that epidermal growth factor (EGF) produced *in vivo* during the process of tissue repair supports the growth of *M*. *avium* and *M*. *tuberculosis*. The researchers determined that EGF binds specifically to mycobacteria with a K_D_ of 2.0x10^-10^ M and an average of 450±60 receptors per cell of *M*. *avium*. Moreover, similarly to our results with IL-8 and *Mtb*, the prior exposure of *M*. *avium* to EGF resulted in an increase in the number of intracellular CFUs in infected macrophages.

In this manuscript, we present *Mtb* AtsG arylsulphatase, SahH hydrolase and GlmU bifunctional transferase as tubercle bacilli proteins engaged in human IL-8 chemokine binding and modulation of pathogen entry into host neutrophils. Taking into account, the expanded attachment/entry of the AtsG-, GlmU- and SahH-overproducing *Mtb* strains into neutrophils and the broad repertoire of PRMs expressed by neutrophils, the findings suggest that the mycobacterial AtsG, GlmU and SahH proteins could represent PAMPs recognized by the yet-to-be-discovered members of the phagocyte PRMs. The discovered interplay between the pathogen and the host phagocytes may have various, positive as well as negative, outcomes for the infected individual and could contribute, on the one hand, to effective pathogen elimination by cells of the innate immune system or, on the other hand, to colonization of the host tissues by the invading mycobacteria. Although manipulation of the host defense elements by *Mtb* is well known and widely described, we are still far from having a full understanding and explanation of the plethora of pathogenic mechanisms used by bacilli to subvert the bactericidal weapons of phagocytes and to induce granuloma formation for achieving long-term residence in the host. In addition, further studies are also necessary to clarify the molecular aspects of the interactions of the mycobacterial AtsG, GlmU and SahH enzymes with the human IL-8 chemokine, with the immunocompetent cells of the innate system and with airway epithelial cells.

## Supporting Information

S1 Table IL-8 binding proteins and peptides list identified by LC/MS/MS. (XLSX)
